# Disorientation patterns of loggerhead sea turtle (*Caretta caretta*) hatchlings in Pinellas County, Florida, USA

**DOI:** 10.1371/journal.pone.0347104

**Published:** 2026-04-15

**Authors:** Kerry L. McNally, Carly Oakley, Megan Davila, Sarah Farinelli

**Affiliations:** 1 Clearwater Marine Aquarium Research Institute, Clearwater Marine Aquarium, Clearwater, Florida, United States of America; 2 Sea Turtle Conservation Program, Clearwater Marine Aquarium, Clearwater, Florida, United States of America; MARE – Marine and Environmental Sciences Centre, PORTUGAL

## Abstract

The disorientation of loggerhead sea turtle (*Caretta caretta*) hatchlings, primarily caused by artificial lighting, poses a significant threat to their survival, as they rely on environmental cues to reach the ocean. In this study, we analyzed data from nesting surveys and disorientation reports to identify factors contributing to disorientation events, including position on the beach and spatial location. Between 2018 and 2023, 1048 nests successfully had hatchlings emerge, with 377 (36%) of these emergences resulting in disorientation events. Nests located in the upper portion of the beach were significantly less likely to result in disoriented hatchlings compared to the middle portion. Spatiotemporal analysis identified areas of hot and cold spots of disoriented hatchlings across different beaches in Pinellas County, Florida, USA, with significant spatial variations of disorientations across years, with 2022 having peaks in spatial clustering and 2021 and 2023 having no clustering. Moonlight was found to play a mitigating role, with significantly more disorientation events occurring on nights with lower moonlight exposure. These findings underscore the need for improved lighting regulations and beach management strategies, such as enhancing natural dunes and beach profiles to reduce artificial light exposure in Pinellas County.

## Introduction

Successful orientation from the nest to ocean is a crucial and vulnerable stage in the life cycle of loggerhead sea turtle (*Caretta caretta*) hatchlings [1]. Hatchlings rely on specific environmental cues to find their way to the ocean, a process known as “sea-finding” [2,3]. These cues include light intensity and wavelength, background illumination, wave reflection, moonlight, and landward silhouettes [2,4–8]. Phototaxis, or the tendency of organisms to move toward or away from light, plays a significant role in how hatchlings orient themselves during their initial crawl to the water [4,5,9–11]. Hatchlings orient toward short wavelength light (<560 nm) such as near-ultraviolet, violet, and blue-green light, and away from longer wavelengths (>560 nm), including green-yellow to yellow-orange light [4]. While phototaxis plays an important role, hatchlings also rely on shape cues, often moving away from elevated silhouettes rather than responding only to light [2,12,13]. Dark silhouettes, such as dunes, create a visual contrast against the seaward horizon, helping to direct hatchlings toward the ocean [7,14].

Disorientation of hatchlings occurs when individuals stray from their natural path to the sea, sometimes moving toward roads, parking lots, and buildings, instead of reaching the ocean [15–18]. As a result, disoriented hatchlings may experience delays or fail to reach the water entirely, increasing their risk of predation, dehydration, and mortality [18]. One of the main causes of disorientation is artificial light along developed coastline, which disrupts the natural navigation process [16,19]. Artificial lights obscure the seaward horizon, a key visual cue, making it harder for hatchlings to distinguish the correct path to the ocean [7,19–21]. Lights from development can attract hatchlings inland by creating a light-trapping effect that increases disorientation risk [7]. Artificial lights can also disorient hatchlings after they enter the water [8,17,22,23]. However, other cues, such as wave direction and magnetic fields, may play a larger role in guiding offshore migration [24,25]. Understanding the effects of light pollution on disorientation is crucial for informing effective mitigation strategies and improving sea turtle conservation outcomes [7,17,18,26].

Beyond individual nests, spatial clustering of disorientation events can provide insight into where mitigation efforts can be most effective. Hot spot analyses, including Optimized Hot Spot Analysis (OHSA) and related spatial clustering methods, have been increasingly used to support conservation decision-making. For example, these approaches have been applied to identify hot spots of large wildfires in Washington State, enabling managers to better target fire-prevention and mitigation efforts [27]; to detect hot and cold spots of forest cover loss in Sri Lanka to guide conflict-mitigation efforts after demonstrating that forest loss was significantly associated with human-elephant conflict [28]; to map pheasant (*Phasianus colchicus*) productivity hot and cold spots in South Dakota to inform targeted habitat management efforts [29]; and to identify overlapping hot spots of jellyfish biomass and leatherback sea turtle (*Dermochelys coriacea*) density in Canadian Atlantic waters, clarifying critical foraging habitat for this endangered species and supporting dynamic, climate-responsive management actions [30].

Moonlight is an important factor in the navigation of hatchlings during their crawl from the nest to the ocean [7,8]. During nights with a full moon, the extra natural illumination may reduce the contrast between artificial light sources and the seaward horizon, potentially decreasing the attractiveness of artificial lights and lowering the likelihood of disorientation [7,12,19,21]. In contrast, during a new moon or when moonlight is obscured, hatchlings are more likely to be drawn to artificial light sources, increasing the risk of disorientation [6,13,16,21,31]. Studies have shown that the presence of moonlight can either mitigate or exacerbate the effects of artificial lighting, influencing hatchlings’ ability to orient themselves correctly [8,32]. Understanding the role of moonlight in sea-finding behavior is important for assessing disorientation risks and developing conservation strategies to reduce light pollution and enhance hatchling survival.

Central West Florida is one of the fastest growing loggerhead sea turtle nesting regions in Florida [1,33]. Pinellas County lies at the northern most end of this nesting range and is characterized by extensive coastal development and high levels of artificial lighting [34]. Natural dunes are limited in the county, and beach morphology varies year to year due to natural disturbances, such as hurricanes and other storm events [35]. The intense artificial light, limited dunes, and dynamic beach profiles make Pinellas County an area of concern for hatchling disorientation.

To reduce the impacts of coastal lighting, the Florida Fish and Wildlife Conservation Commission (FWC) established criteria for “turtle-friendly” lighting, recommending well-shielded, downward-directed fixtures that emit long-wavelength light (560 nm or greater, such as amber or red) to reduce hatchling disruption [36]. These guidelines form the basis for lighting ordinances, which specify the appropriate time frames, approved light fixtures, wavelengths, and shielding requirements to minimize lighting's impact on sea turtles [37]. Despite these guidelines, adoption of “turtle-friendly” lighting remains limited by residents and commercial property owners. Even filtered or amber LED lighting, although less attractive than white lights, can still disrupt orientation [31,32,38,39], which reinforces the need for shielding, placement, and enforcement to effectively protect hatchlings [39].

The objectives of this study were to examine the extent of disorientations in loggerhead sea turtle hatchlings in Pinellas County, Florida, USA, from 2018 to 2023, and to identify the environmental factors and spatiotemporal patterns associated with both the occurrence and severity of these events. We hypothesized that disorientation probability and severity would vary in relation to nest position on the beach, with higher rates occurring on beach locations more exposed to upland artificial lighting. We further expected that disorientation patterns would exhibit spatial clustering. To address this objective, we integrated OHSA to identify statistically significant clusters of elevated disorientation rates (hot spots) and clusters where disorientation rates are significantly lower than expected (cold spots). By analyzing disorientation patterns, we aim to inform lighting mitigation and beach management strategies and contribute to the conservation of sea turtles in the region.

## Methods

### Nesting surveys

**Fig 1:**
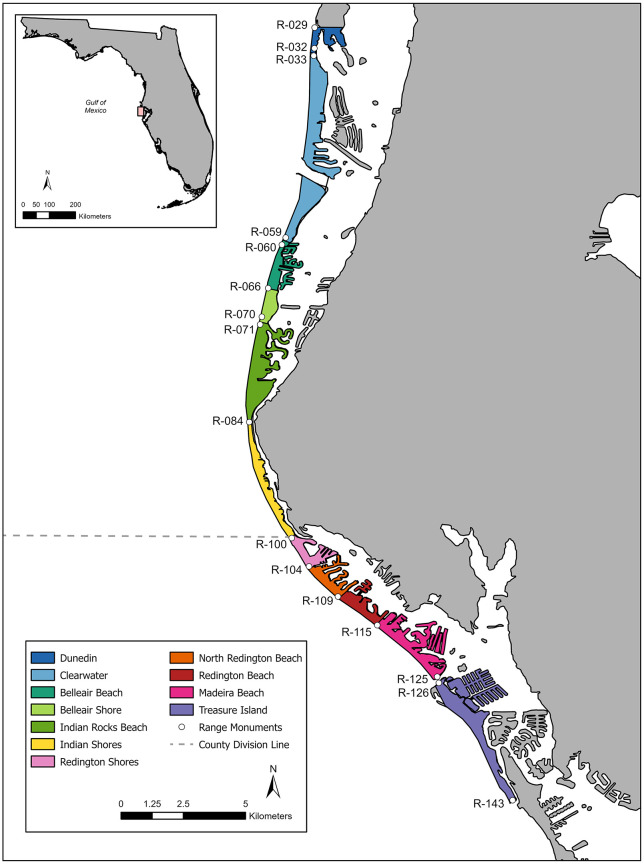
Study area. Map depicting eleven municipalities that make up north and central Pinellas County, Florida, USA, surveyed by Clearwater Marine Aquarium for loggerhead sea turtle nesting activity. The white dots identify the coastal range monuments. The dashed line indicates the division of north Pinellas County (surveyed under MTP #263), and central Pinellas County (surveyed under MTP #013). The basemap is the Detailed Florida State Boundary from the Florida Department of Transportation Open Data Portal (https://gis-fdot.opendata.arcgis.com/datasets/fdot::detailed-florida-state-boundary/about). FDOT GIS data are publicly available and distributed for open use; Cartographic software: ArcGIS. Coastal range monument data were obtained from the Florida Department of Environmental Protection Open Data Portal (https://ca.dep.state.fl.us/arcgis/rest/services/OpenData/COASTAL_ENV_PERM/MapServer/10).

Since 1990, Clearwater Marine Aquarium (CMA) conducted nesting surveys on Gulf of Mexico beaches in Pinellas County, Florida, USA under Marine Turtle Permits #263 (north Pinellas County) and #013 (central Pinellas County) issued by the Florida Fish and Wildlife Conservation Commission (FWC) (Fig 1). CMA surveyed north Pinellas County in all years; central Pinellas County was surveyed only in years when Permit #013 was held. As a result, CMA did not hold Permit #013 in 2018 and 2019 so we did not collect data in central Pinellas County during these two years. As a whole, north and central Pinellas County includes 33.59 km of barrier island beaches consisting of 11 different municipalities. North Pinellas County ranges from coastal range monument R-028 at Dunedin Pass (28.01879 N, 82.82649 W) south to R-100 in Indian Shores (27.83804 N, 82.83857 W), with the municipalities of Dunedin, Clearwater, Belleair Beach, Belleair Shore, and Indian Rocks Beach in between (Fig 1). Central Pinellas County ranges from R-100 to R-143 in the south end of Treasure Island (27.73869 N, 82.75641 W), with the municipalities Redington Shores, North Redington Beach, Redington Beach, and Madeira Beach in between (Fig 1).

Nesting surveys were conducted daily from April 15th to October 31st, annually. Morning surveys began as early as 30 minutes before sunrise by driving along the shoreline to identify the occurrence of female sea turtles crawling up the beach to lay a nest. All observed nests were marked and monitored daily according to the FWC Marine Turtle Conservation Handbook [40]. During the surveys, CMA personnel also identified evidence of hatchling emergence and collected data accordingly.

### Data collection

A FWC consent permit and data management agreement authorized the use of FWC nest and disorientation data collected during permitted survey and monitoring programs for this study. We excluded data collected prior to 2018 from this study because restraining cages were often used to protect the hatchlings from predators, which prevented determining the direction in which the hatchlings would have emerged from the nest and whether a disorientation occurred. Data collected from nesting activities by loggerhead sea turtles from 2018 to 2023 included those reported in McNally et al 2024 [41], which included GPS location, distance to nearest upland barrier, beach width (distance from the high tide line to the upland barrier), position of the beach (lower, middle, or upper third of the beach), number of emerged hatchlings (quantified by the number of hatched eggs in a nest), and clutch size (quantified as the summation of hatched, pipped, unhatched, and damaged eggs, as was determined from final nest inventories conducted at least 72 hours after emergence).

Nest emergence was determined based on the presence of four or more hatchling crawl tracks on the beach or nest inventory data indicating hatched shells [40]. A nest emergence was classified as a disorientation event when tracks of a minimum of five hatchlings showed disorientation [40]. FWC Marine Turtle Disorientation Reports were completed as part of the FWC Disorientation Monitoring Program for each event. Data collected for disorientation reports include GPS location, municipality, and whether any disoriented turtles eventually reached the water.

The data also included any protective measures applied to reduce disorientation, if applicable. Although rarely, the only protective measure used in Pinellas County during this study period was a light barrier. A light barrier is any device used to block anthropogenic light from a nest, preventing light pollution from affecting the emerging hatchlings. The goal of the barrier is to help hatchlings orient toward the water without causing disorientation [40].

CMA personnel also collected further details on the disorientation events included in this study, such as orientation direction and the environment or surface the hatchlings moved toward. Through these additional data, we were able to determine the total number of hatchlings that were disoriented from a nest, including how many were oriented towards areas of sandy beach, the dunes, road or parking lot, pathway, pools, storm drains, or yards/patios. For each nest, we quantified the number of hatchling tracks that appeared to reach the water and the number of hatchlings found deceased. Because these data are based on observed tracks, the number of disoriented hatchlings is estimated in cases where tracks were obscured from human traffic or weather events. We also calculated the percentage of emerged hatchlings that disoriented by dividing the number of disoriented hatchlings by the total number of emerged hatchlings per nest.

### Statistical analysis

We analyzed data from 2018 to 2023 in R Program Version 4.3.0 [42]. We only used data from nests that had evidence of emergence, as disorientation can only be assessed when hatchlings leave the nest. To assess the influence of nest-specific, spatiotemporal, and environmental variables, including beach width, distance from the upland barrier, beach position, month, and year, we used two approaches. First, we fit generalized linear mixed models (GLMMs) with a binomial distribution to evaluate the occurrence (presence or absence) of disorientation using the glmmTMB package (version 1.1.11) in R. To further evaluate the severity of a disorientation, defined as the proportion of disoriented hatchlings that emerged from a nest, we then fit beta-binomial GLMMs. Five nests were excluded from the analysis of severity because the percentage of emerged hatchlings that disoriented was unknown. We initially evaluated municipality as a random effect, but it was ultimately excluded because several municipalities contained very few nests, resulting in unstable model estimates. Because of this, the final model is equivalent to a GLM. To evaluate short-term directional trends, year was included as a numeric predictor. When significant differences were detected, we performed pairwise comparisons using estimated marginal means with the emmeans function in R [43], with Benjamini-Hochberg adjustments to control for multiple comparisons [44]. Model fit was assessed using simulated residuals with the DHARMa package in R [45], including tests for residual uniformity, dispersion, and zero inflation. Statistical significance was set at p ≤ 0.05.

### Hot spot analysis

Disorientation events from 1043 nests with a known percentage of emerged hatchlings across all study years (2018–2023) were imported into ArcGIS Pro Version 3.3.0 [46], and sample size varied by year (Table 1). These point data were projected in the coordinate system for Florida (WGS 1984 UTM 17N). An Optimized Hot Spot Analysis (Getis-Ord Gi*) (OHSA) was independently performed for each year using the Spatial Statistics tool to identify spatial clustering of hatchling disorientation severity. This analysis identified nests with significantly higher or lower proportions of emerged hatchlings that were disoriented, which were designated as hot spots and cold spots, respectively.

**Table 1:** Sample size (number of emerged nests) and distance bands used for Optimized Hot Spot Analysis (OHSA) by year (2018–2023), and the percentage of nests affected by disorientation. A fixed distance band was applied for all years except 2021, which used the k-nearest neighbors method (k = 6). Sample size was determined by the number of nests that had a known percentage of hatchings emerge, which excluded 5 nests from the full sample set (2 from 2023, 2 from 2022, and 1 from 2021).

Year	Emerged nests	Distance band (m)	% nests disoriented
2018	164	720	26
2019	175	291	35
2020	154	704	28
2021	134	–	29
2022	269	830	43
2023	147	620	50

In an OHSA, each point is a feature that is assigned a value (i.e., percentage of hatchlings that emerged from a nest and disoriented), and every feature is assigned to a neighborhood. The tool defines neighborhoods based on the proximity of the point data, where each point is evaluated relative to its surrounding points within a given distance band that was optimized to best capture local spatial patterns of clustering. For all OHSAs, distance bands were first calculated using the Fixed Distance Band method, which uses incremental spatial autocorrelation to determine the distance at which the clustering is most significant, as indicated by the distance with the greatest z-score (Table 1). If an optimal distance is not able to be determined using this method, the tool then uses the K-Nearest Neighbors method, which selects an optimal distance based on the spatial distribution of features. The tool also removes all outliers prior to computing the optimal distance. Fixed Distance Band was used to calculate the optimal distances for all years except for 2021, which used the K-Nearest Neighbors method with k equal to 6 (Table 1).

Once the distance bands are calculated, the tool determines whether the values in each neighborhood are significantly different from the entire study area (i.e., all nest locations) and corrects for multiple testing and spatial dependence with the False Discovery Rate (FDR) correction method. If a neighborhood has significantly higher values compared to the study area, all features of that neighborhood are marked as a statistically significant hot spot (i.e., high percentage of disoriented hatchlings). Conversely, if a neighborhood has significantly lower values compared to the study area, all features of that neighborhood are marked as a statistically significant cold spot (i.e., low percentage of disoriented hatchlings). The output is a new feature class with a z-score, p-value, and confidence level. Features with high z-scores and small p-values are statistically significant hot spots, whereas features with low, negative z-scores and small p-values are statistically significant cold spots. The absolute value of the z-score indicates the strength of the clustering intensity [47,48]. This statistical approach allows for the identification of hot and cold spots at various levels of significance (90%, 95%, and 99%).

If there were statistically significant (95% and 99% confidence levels) hot and cold spots in a majority of municipalities, a chi-square test of independence was performed in R to determine whether the distribution of hot and cold spots was independent of municipality. If the data violated the assumption that all expected cell frequencies in the matrix are ≥ 5, a Monte Carlo simulation with 10,000 iterations was used to estimate the p-value. Additionally, Pearson residuals and their contributions to the total chi-square statistic were examined to help interpret which municipalities were associated most to deviations from the expected distribution of hot or cold spot frequencies.

### Moonlight

Using the moonlit package in R [49], we calculated the moon phase (or the fraction of the moon illuminated, where 1 represents a full moon and 0 represents a new moon) for each date a nest hatched. We then performed a Wilcoxon Rank-Sum Test to determine whether disorientation events were more likely to occur based on the fraction of the moon illuminated. We also conducted Spearman correlation to examine the relationship between the fraction of the moon illuminated and the percentage of hatchlings from each nest that were disoriented.

## Results

### Disorientation data

From 2018 to 2023, 1479 loggerhead sea turtle nests were recorded in north and central Pinellas County. Of these, 1048 nests had hatchlings emerge, with 36% (377) resulting in hatchling disorientation events (Table 1). The annual mean percentage of nests that resulted in disorientations was 35 ± 10% (range 26–50%). Among nests that experienced disorientation, the proportion of emerged hatchlings that were disoriented averaged 54 ± 31% (range 4–100%). Out of 377 disorientation events, 39% of the disoriented hatchlings reached the water, averaging 15 ± 16% (range 0–85%) per nest. Of the total disoriented hatchlings, 6% reached roads and parking lots, averaging 2 ± 8 (range 0–84) hatchlings per disorientation event. The remaining hatchlings reached sandy areas, dunes, yards, or walking paths based on the direction of their tracks. Out of 1048 nests, 23 were equipped with light barriers. Out of the 377 disorientation events recorded, 5 nests were equipped with light barriers.

**Fig 2:**
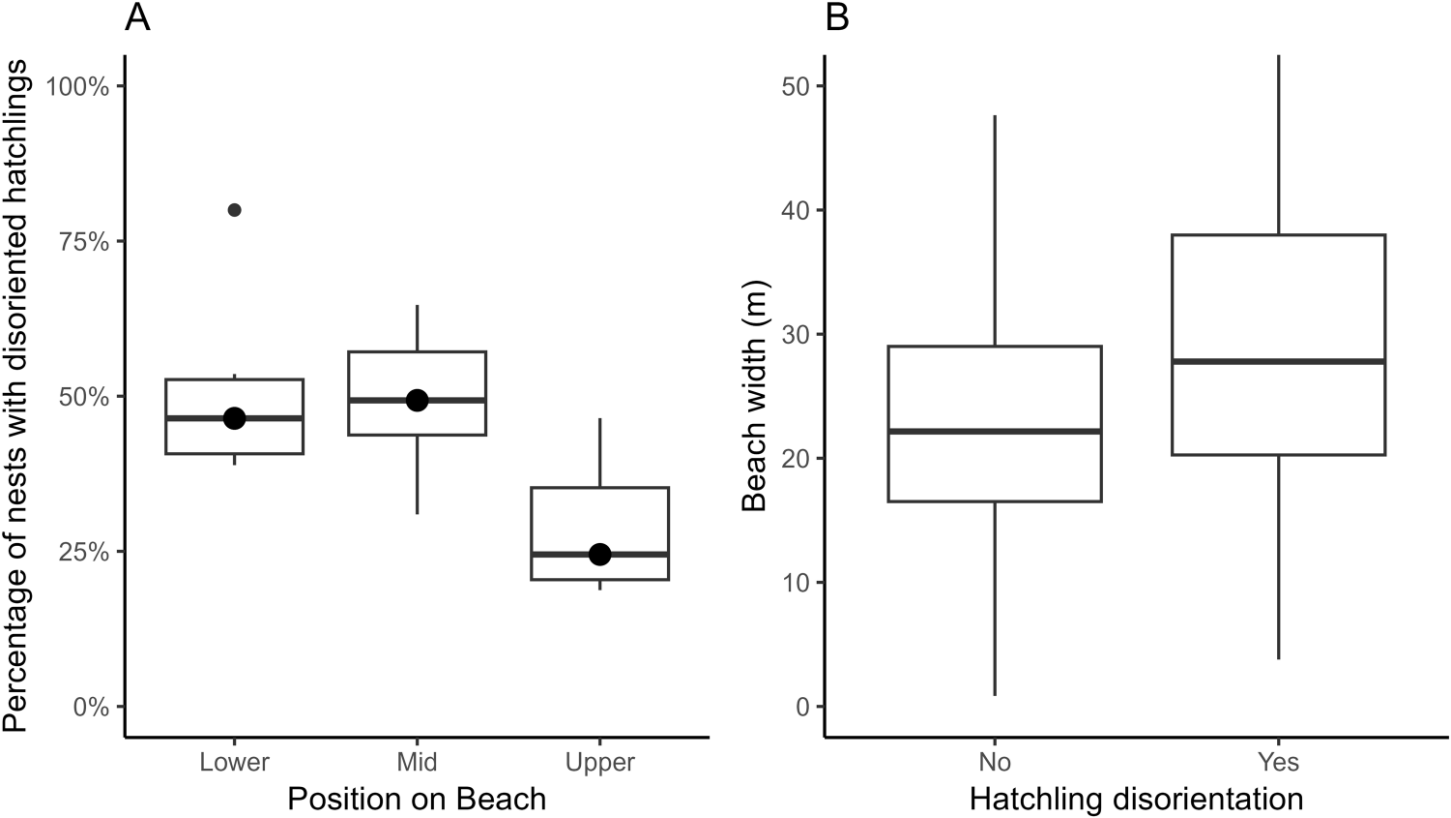
Boxplots of the percentage of nests that disoriented. **(A)** the annual percentages of nests that disoriented at each position on the beach. **(B)** Relationship between the beach width and occurrence of disoriented nests. Boxplots display the median, interquartile range, and range of annual percentages.

The annual mean percent of nests laid on the lower position of the beach that disoriented was 51 ± 15% (range 39–80%, median = 46%, n = 40), 49 ± 12% (range 31–65%, median = 49%, n = 124) for the middle position, and 29 ± 11% (range 19–47%, median = 35%, n = 213) for the upper position (Fig 2). Nests laid on the upper position of the beach had a significantly lower probability of disorientation compared to the mid beach position (estimate = 0.83, Z = 4.29, p < 0.001), but did not differ significantly from the lower beach position (estimate = 0.58, Z = 1.32, p = 0.18) (Fig 2A). A similar pattern was observed for disorientation severity, with a lower proportion of emerged hatchlings disorienting at the upper position compared to the mid position (estimate = 0.78, Z = 4.77, p < 0.001).

On wider beaches, there was an increased probability of a disorientation event (estimate = 0.04, Z = 4.34, p < 0.001; Fig 2B), as well as disorientation severity (estimate = 0.03, Z = 4.11, p < 0.001). Although disoriented nests were, on average, located farther from the upland barrier (S1 Table), this difference largely reflects variation in nest placement across beach positions. After accounting for covariates, the greater distance from the upland barrier actually associated with a lower probability of disorientation (estimate = −0.02, Z = −2.25, p = 0.025). However, distance from the upland barrier was not significantly associated with disorientation severity (estimate = −0.01, Z = −1.15, p = 0.251).

Year-specific variation was evident when year was modeled as a categorical factor (Tables 1 and S2). When year was included as a continuous predictor, a short-term directional increase was detected in both the probability of disorientation (estimate = 0.26, Z = 5.89, *p* < 0.001) and disorientation severity (estimate = 0.27, Z = 7.08, *p* < 0.001). Seasonal timing, indicated by month, was not significantly associated with disorientation probability or severity. The annual mean percent of nests laid in May that disoriented was 36 ± 15% (range 17–62%), 35 ± 11% (range 22–50%) for June, and 32 ± 11% (range 21–50%) for July.

### Hot spot analysis

**Fig 3:**
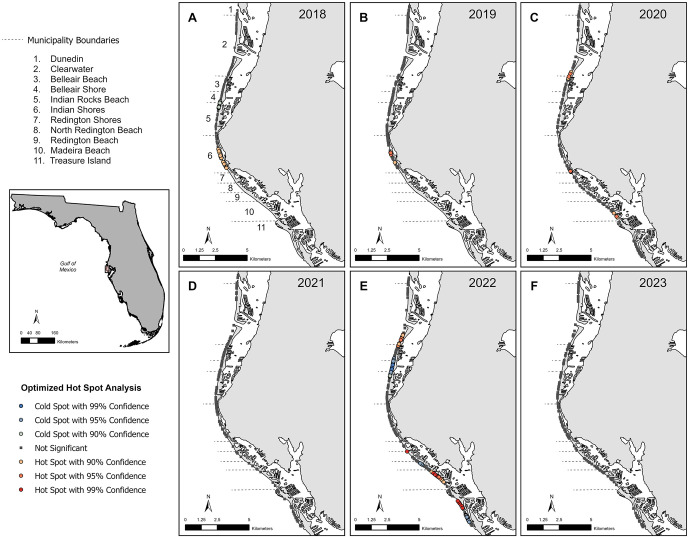
Results of Optimized Hot Spot Analyses (OHSA) of hatchling disorientation along the Gulf coastline of Pinellas County, Florida. Panels A-F show annual hot and cold spots of the percentage of hatchlings that emerged and subsequently disoriented from 2018 to 2023. Disorientation data were not available for central Pinellas County in 2018 and 2019 (Panels A and B), resulting in reduced spatial coverage in those years. Statistically significant hot spots (higher than expected values) are shown in shades of red, and cold spots (lower than expected values) are shown in shades of blue, at 90%, 95%, and 99% confidence levels. Gray squares indicate areas with no statistically significant clustering. An inset map shows the location of the study area on the west coast of Florida. The basemap is the Detailed Florida State Boundary from the Florida Department of Transportation Open Data Portal (https://gis-fdot.opendata.arcgis.com/datasets/fdot::detailed-florida-state-boundary/about). FDOT GIS data are publicly available and distributed for open use; Cartographic software: ArcGIS.

**Fig 4:**
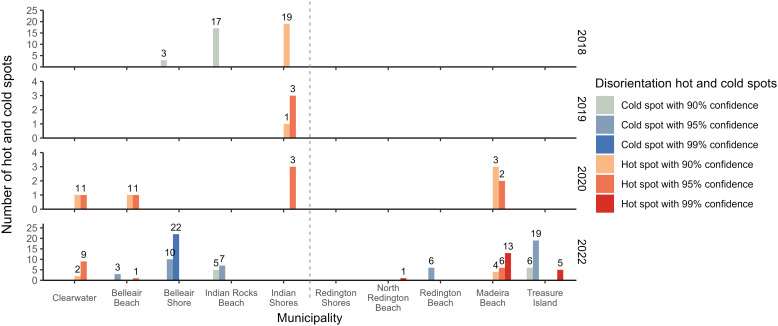
Number of hot and cold spots for each year. The dashed line indicates where north Pinellas County ends and central Pinellas County begins. Only data from north Pinellas County was collected in 2018 and 2019, so the lack of hot or cold spots in those municipalities are due to the lack of data. Years 2021 and 2023 had no hot or cold spots so are not shown in the graph.

The OHSA revealed clear spatiotemporal patterns in hatchling disorientation clustering across Pinellas County (Fig 3). In the earliest survey years (2018 and 2019), when effort was limited to north Pinellas County, clustering was relatively localized (Figs 3A, 3B and 4). Indian Shores consistently exhibited hot spots during both years, while cold spots were detected only in 2018 at Indian Rocks Beach and Belleair Shore (Figs 3A, 3B and 4). Beginning in 2020, clustering became more widespread, with hot spots emerging across four municipalities (Clearwater, Belleair Beach, Indian Shores, and Madeira Beach) (Figs 3C and 4). Notably, 2019 and 2020 exhibited only hot spots (Figs 3B, 3C and 4), whereas both 2018 and 2022 demonstrated a mix of hot and cold spot clustering (Figs 3A, 3E and 4). No significant hot or cold spots occurred in 2021 (Fig 3D) or 2023 (Fig 3F). Spatial clustering peaked in 2022, both in magnitude and distribution, with extensive hot spots concentrated in Madeira Beach and Clearwater, and cold spots strongly centered in Belleair Shore and Treasure Island (Figs 3E and 4). A Chi-square test of independence revealed a significant association between the frequency of disorientation hot spots, cold spots, and non-significant spots and municipalities (χ² = 243.98, p < 0.001). Examination of the Pearson residuals and their contributions to the overall Chi-square statistic indicated that Belleair Shore had the strongest positive association with cold spot frequency (31%), while Madeira Beach showed the strongest positive association with hot spot frequency (20%).

When results were examined collectively across years, consistent spatial trends emerged: Belleair Shore, Indian Rocks Beach, and Redington Beach consistently exhibited only cold spots, whereas Clearwater, Indian Shores, North Redington Beach, and Madeira Beach consistently exhibited only hot spots (Figs 3 and 4). In contrast, Belleair Beach and North Redington Beach had comparatively few hotspots overall, and Dunedin and Redington Shores never exhibited significant clustering in any survey year (Figs 3 and 4).

### Moonlight

**Fig 5:**
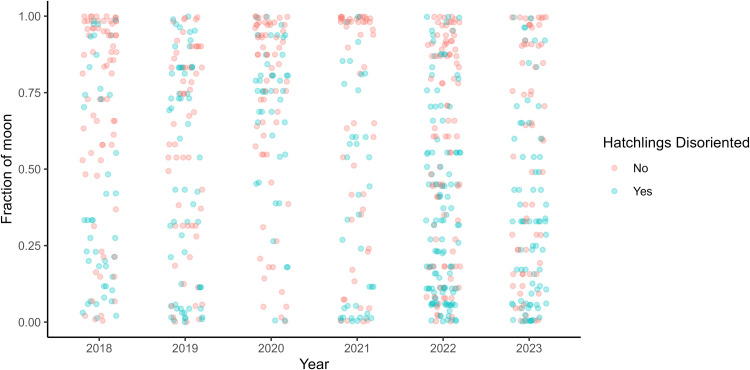
Jitter plot illustrating the relationship between moon fraction for each year a nest hatched and whether hatchlings disoriented. A full moon is represented by a moon fraction of 1, and 0 represents a new moon. Each point represents a nest, showing variability in disorientation occurrence across different moon phases.

The mean lunar fraction was significantly higher during non-disorientation events (0.6 ± 0.3) than during disorientation events (0.4 ± 0.3), indicating that disorientations were more common on darker nights (W = 98700, p < 0.001). The proportion of emerged hatchlings that disoriented also increased with decreased moonlight (ρ = −0.27, S = 944304, p < 0.001) (Fig 5).

## Discussion

The results from our analysis indicate that hatchling disorientation is shaped by multiple environmental variables in Pinellas County. As observed in other studies, the majority of disorientation events involved at least some hatchlings successfully reaching the water [19,50]. Specifically, our study found that 39% of disoriented hatchlings managed to reach the ocean. The remaining 61%, particularly those that ended up on roads or parking lots, are generally presumed to have died [15,50,51]. Previous studies in Florida have highlighted the risks faced by disoriented hatchlings in urban environments, such as roads and parking lots, which significantly increase mortality rates [7,51]. Because tracks are estimates and many cross areas with human traffic or are washed out by tides, the number of hatchlings reaching the water is likely underestimated in our study.

Hatchlings from nests located on the upper position of the beach were less likely to become disoriented than those from nests on the mid position. This pattern aligns with findings from other regions in Florida, where nests closer to the dunes tend to experience less disorientation [6,14]. For those nests where hatchlings did disorient, a smaller proportion of emerged hatchlings disoriented in the upper position of the beach compared to the mid position. The dune silhouette of the upper beach can partially block or reduce visibility of upland artificial lighting, playing a critical role in limiting disorientation because hatchlings typically orient towards the lower, brighter horizon [2,6,9,12]. The mid position of the beach is more exposed to the artificial light from development and there is less contrast between horizons, increasing the susceptibility of hatchlings to disorient. The lack of a significantly higher disorientation rate for nests on the lower position relative to the mid and upper may appear counterintuitive, but hatchlings emerging closer to the ocean may rely more strongly on the ocean horizon, which can partially offset the influence of landward light sources [52]. The mean percentage of disoriented nests was very similar between the lower and mid positions, indicating a small effect size, and the small sample size of lower position nests limits inference for this position and may bias comparisons.

Consistent with our results of beach position, wider beaches were associated with both a high probability of occurrence and severity of disorientations. On wider beaches, hatchlings must travel longer distances to the ocean, increasing the exposure time to artificial light. Contrary to expectations, the greater distance from the upland barrier was associated with a lower probability of disorientation [14]. This may reflect that the proximity to the ocean horizon also plays a role in orientation [52]. However, once a disorientation did occur, the distance from the upland barrier did not significantly influence the severity of disorientation, which suggests that factors driving the initial disorientation may differ from those influencing the proportion of hatchlings disorienting within a nest. In Pinellas County, the lack of substantial dunes likely contributes to the high rates of disorientation even among upper beach nests. Without prominent dunes, the natural horizon cues are less effective because dunes normally block artificial lighting from upland sources and create a clear path to the ocean horizon, which hatchlings use for orientation [2,13,14,20]. In Pinellas County, low dune profiles and frequent erosion from storm events [35], reduce these protective measures [53].

Our results highlight the need to incorporate mitigation strategies that combine the reduction of artificial light and increasing the height of upland barriers such as dunes to reestablish natural dune silhouettes [14,20,54–56]. However, building up dunes may attract predators to the vegetation and face opposition from residents concerned about obstructed ocean views [52,54]. Despite these challenges, dune restoration and maintenance remain viable management approaches in Pinellas County where there are limited natural dunes, with many having been destroyed or developed. In response to the lack of natural dunes in Pinellas County, artificial dunes have been constructed in several areas, including Clearwater Beach, Treasure Island, and Madeira Beach [57,58]. After Hurricane Idalia caused significant erosion in 2023, the county launched an emergency shoreline restoration project to stabilize the coastline [58]. While the primary goals of dune restoration in the county are to protect property and maintain beach integrity, these efforts may also help reduce hatchling disorientation. Future data collection could include detailed information of dune presence and characteristics in conjunction with information on the artificial light (type, wavelength, brightness) at individual nest sites to allow for evaluation of their influence on hatchling disorientation. Continued monitoring of hatchling disorientation events will also be critical in assessing the long-term impact of lighting ordinances and dune restoration, particularly as storm activity continues to reshape beach profiles and potentially limit the durability of these interventions. Site-level monitoring before and after the next scheduled dune restoration project could also offer valuable insight.

Our analysis also shows a short-term increase in the rate of disorientation as well as severity over the six-year study period. This trend is concerning given that disorientation rates in Pinellas County (36%) far exceed the 2018 statewide mean of 3% reported for Florida [54]. Although long-term trends cannot be inferred from a six-year time period, the consistent directional increase may reflect changes in lighting intensity and beach morphology associated with erosion. Our analysis revealed no significant differences in disorientation rates across the different months of the nesting season. This lack of monthly variation in disorientation rates suggests that environmental factors that could cause disorientation, such as human activity and artificial lighting, remained relatively stable throughout the season. This supports the need for continuous mitigation and enforcement throughout the nesting season rather than month-specific management.

Light barriers, a mitigation strategy to block artificial light from the hatchlings, can enhance the safety of urban beaches for emerging hatchlings. In our dataset, 23 nests were treated with light barriers, with 19% of those resulting in disoriented hatchlings. Previous research suggests that low or incomplete light barriers may still allow diffuse light to influence hatchling orientation [55], which could explain why some disorientation events occurred despite the use of these barriers in our study. Due to limited usage of light barriers in Pinellas County, we could not make conclusions about its effectiveness, but it is important to note that there are limitations to their use if not installed correctly.

Our OHSA indicated that hatchling disorientation deviated from a random or uniform spatial distribution, forming statistically significant clusters in several years across municipalities. The presence of disorientation hot spots in municipalities such as Clearwater Beach, Treasure Island, Indian Shores, and Madeira Beach was not unexpected given their known levels of coastal development and artificial lighting (Oakley, personal observation). However, the specific locations and intensity of disorientation varied by year. One possible reason is the changes in coastal development and population. Another possible reason for the variability is storm events, which are known to reshape beaches, and cumulative impacts of multiple storm years may progressively erode dune systems, exacerbating lighting exposure [57,59]. Storms in the early seasons of years, such as 2021 (Tropical Storm Elsa in July 2021) and 2023 (meteotsunami in June 2023) affect the emergence of nests already laid, which may potentially lead to changes in patterns of hot spots and/or cold spots (https://www.weather.gov/tbw/tbwweatherevent). Notably, 2021 and 2023 had the smallest sample sizes of all study years (i.e., number of emerged nests), and these were also the only years in which no statistically significant hot or cold spots were detected. Storm-related nest loss may result in fewer remaining nests with disoriented hatchlings, reducing the sample size and thereby limiting the statistical power to detect spatial clustering through OHSA. In addition to reducing the statistical power, a lower number of nests can limit spatial coverage and weaken the spatial autocorrelation required for hot spot detection, potentially resulting in a more diffuse or random distribution that obscures true clustering patterns. Another possibility is changes in storm damage or in property ownership with periods of compliance to lighting ordinances. After a major storm, beach lighting or human activity may also be more uniformly low across the area due to power outages or reduced tourism, leading to less variation in disorientation risk and therefore less clustering.

The greatest number of hot and cold spots were identified in 2022, which also had the highest number of emerged nests compared to other years. This larger sample size may have improved the ability to detect these spatial patterns, possibly explaining shifts from previous years. For example, Indian Shores had hot spots in earlier years but none in 2022. In 2022, cold spots were found in Belleair Shore and southern Treasure Island, areas characterized by more vegetation and prominent escarpments or seawalls that likely create darker silhouettes. These areas also primarily consist of single-family homes. In contrast, a hot spot in Madeira Beach occurred in a brightly lit resort area with no escarpment. Redington Beach had no hot or cold spots in any year, likely due to the low number of nests as it averaged only five emerged nests per year during the study period.

As observed in previous studies, full moons are associated with reduced disorientation, while new moons tend to correlate with higher disorientation rates [8,16,32]. This suggests that the presence of moonlight may mitigate the effects of artificial lighting, either by masking it or by providing a natural light source that aids hatchling orientation [7,13,21,60]. Turtle-friendly amber LED lights can also disrupt hatchlings’ ability to find the sea, with the effect being more pronounced on moonless nights [31]. These findings underscore the importance of considering both natural and artificial light sources in conservation strategies. Adaptive lighting management could incorporate lunar cycles by prioritizing lighting compliance and temporary mitigation measures (i.e., dimming or shielding artificial lighting during peak hatching times) during darker nights, especially around new moon phases, when there is a higher risk of disorientation.

## Conclusion

Sea-finding behavior in hatchlings is influenced by the synergistic interactions of multiple environmental cues and sensory inputs [5,19,61,62]. The lack of dune silhouettes and the presence of artificial lighting are two critical factors contributing to disorientation in Pinellas County, as well as their interaction [7,16]. Several studies have recommended combining dune restoration, which enhances natural cues, with efforts to reduce artificial lighting wherever feasible [14,16,54,56]. Such an integrated approach would allow for more comprehensive mitigation strategies, including enforcing light reduction during new moon nights and peak hatchling emergence periods between dusk and dawn [7,60]. Implementing these strategies may be crucial for improving hatchling orientation and ensuring the long-term conservation of sea turtle populations in Pinellas County.

Our findings demonstrate that hatchling disorientation is not randomly distributed but instead exhibits clear spatial clustering, particularly evident in 2022. However, the presence and intensity of hot and cold spots varied across years, highlighting temporal variability that should be considered in management planning. The consistent identification of hot spots in certain municipalities suggests that targeted lighting mitigation and outreach efforts should be prioritized in these areas. It is important to note that the hotspot itself does not necessarily indicate that lighting within the municipality alone is responsible. Nearby municipalities may also contribute to hatchling disorientation, and effective mitigation may require coordination across adjacent areas. Municipalities that consistently exhibit cold spots, such as Belleair Shore, may serve as valuable models for effective lighting management and conservation best practices. Because OHSA identifies spatial clustering rather than overall disorientation rates, years without hot spots may still experience high disorientation (i.e., 2023), reinforcing that OHSA is one tool in the toolbox and should be used alongside complementary metrics.

## Supporting information

S1 Table Summary of nesting survey data. Summary data for all sea turtle nests. Non-disoriented nests and disoriented nests are shown separately to highlight differences in hatchling disorientation patterns. SD = standard deviation; Min = minimum; Max = maximum.
(XLSX)

S2 Table Annual summary of nesting survey data. Summary data for sea turtle nests by year. SD = standard deviation; Min = minimum; Max = maximum.
(XLSX)

S3 Table Summary of nesting data for municipalities. Summary data for sea turtle nests by municipality. Annual data per municipality includes mean ± standard deviation (minimum – maximum). CW = Clearwater Beach and Dunedin; BB = Belleair Beach; BS = Belleair Shore; IRB = Indian Rocks Beach; IS = Indian Shores; RS = Redington Shores; NRB = North Redington Beach; RB = Redington Beach; MB = Madeira Beach; TI = Treasure Island.
(XLSX)

S4 Table Full dataset. Complete dataset of all data analyzed in this study, excluding GPS coordinates of individual nests. Because this data is owned by FWC, a consent permit for these coordinates can be requested through FWC, by contacting MTP@myfwc.com.
(XLSX)
